# Evidence for the effectiveness of chlorhexidine bathing and health care-associated infections among adult intensive care patients: a trial sequential meta-analysis

**DOI:** 10.1186/s12879-018-3521-y

**Published:** 2018-12-19

**Authors:** Steven A. Frost, Yu Chin Hou, Lien Lombardo, Lauren Metcalfe, Joan M. Lynch, Leanne Hunt, Evan Alexandrou, Kathleen Brennan, David Sanchez, Anders Aneman, Martin Christensen

**Affiliations:** 1 Critical Care Research in Collaboration & Evidence Translation (CCRiCET), Sydney, Australia; 2 Centre for Applied Nursing Research, School of Nursing and Midwifery, Western Sydney University and Ingham Institute of Applied Medical Research, Sydney, Australia; 3 Department of Intensive Care, Liverpool Hospital, Sydney, Australia; 4 Department of Intensive Care Bankstown-Lidcombe Hospital, Bankstown, Australia; 5 Department of Intensive Care Campbelltown Hospital, Campbelltown, Australia; 6 South Western Sydney Clinical School, Faculty of Medicine University of New South Wales, Sydney, Australia; 7 Centre for Applied Nursing Research, Ingham Institute of Applied Medical Research, South Western Sydney Local Health District (SWSLHD), Level 3, room 3.45, 1-3 Campbell St Liverpool 2170, Locked Bag 7103, Liverpool BC, Sydney, NSW 1871 Australia

**Keywords:** Intensive care, Preventing hospital acquired infection, Chlorhexidine bathing, Meta-analysis, Trial sequential analysis

## Abstract

**Background:**

Health care associated infections (HAI) among adults admitted to the intensive care unit (ICU) have been shown to increase length of stay, the cost of care, and in some cases increased the risk of hospital death (Kaye et al., J Am Geriatr Soc 62:306-11, 2014; Roberts et al., Med Care 48:1026-35, 2010; Warren et al., Crit Care Med 34:2084-9, 2006; Zimlichman et al., JAMA Intern Med 173:2039-46, 2013). Daily bathing with chlorhexidine gluconate (CHG) has been shown to decrease the risk of infection in the ICU (Loveday et al., J Hosp Infect 86:S1-S70, 2014). However, due to varying quality of published studies, and varying estimates of effectiveness, CHG bathing is not universally practiced. As a result, current opinion of the merit of CHG bathing to reduce hospital acquired infections in the ICU, is divergent, suggesting a state of ‘clinical equipoise’.

This trial sequential meta-analysis aims to explore the current status of evidence for the effectiveness of chlorhexidine (CHG) bathing, in adult intensive care patients, to reduce hospital acquired infections, and address the question: do we need more trials?

**Methods:**

A systematic literature search was undertaken to identify trials assessing the effectiveness of chlorhexidine bathing to reduce risk of infection, among adult intensive care patients. With particular focus on: (1) Blood stream infections (BSI); (2) Central Line Associated Blood Stream Infections (CLABSI); (3) Multi-Resistant Drug Organism (MRDO); (4) Ventilator Associated Pneumonia; and, Catheter Associated Urinary Tract Infections (CAUTI). Only randomised-control or cluster randomised cross-over trials, were include in our analysis. A Trial Sequential Analysis (TSA) was used to describe the current status of evidence for the effectiveness of chlorhexidine (CHG) bathing, in adult intensive care patients, to reduce hospital acquired infections.

**Results:**

Five trials were included in our final analysis - two trials were individual patient randomised-controlled, and the remaining cluster-randomised-crossover trials. Daily bathing with CHG was estimated to reduce BSI in the ICU by approximately 29% (Der-Simonian and Laird, Random-Effects.

(DL-RE) Incidence Rate Ratio (IRR) = 0.71, 95% confidence interval (CI) 0.51, 0.98); reduce CLABSI in the ICU by approximately 40% (DL-RE IRR = 0.60, 95% CI 0.34, 1.04); reduce MDRO in the ICU by approximately 18% (DL-RE IRR = 0.82, 95% CI 0.69, 0.98); no effect in reducing VAP in the ICU (DL-RE IRR = 1.33, 95% CI 0.81, 2.18); and, no effect in reducing CAUTI in the ICU (DL-RE IRR = 0.77, 95% CI 0.52, 1.15). Upper (superiority) monitoring boundaries from TSA were not crossed for all five specific infections in the ICU.

**Conclusion:**

Routine bathing with CHG does not occur in the ICU setting, and TSA suggests that more trials are needed to address the current state of ‘clinical equipoise’. Ideally these studies would be conducted among a diverse group of ICU patients, and to the highest standard to ensure generalisability of results.

## Background

Health care associated infections among adults admitted to the intensive care, have been shown to increase length of stay, the cost of care, and in some cases an increased risk of hospital death [1–4]. Daily bathing with chlorhexidine gluconate has been suggested as an effective intervention to reduce the risk of infection during an intensive care stay [5–8]. However, the effectiveness of CHG bathing to reduce ICU infections has varied considerably among published trials, making the effectiveness of CHG bathing in ICU patients uncertain [9], and possibly reliant on the underlying risk among the given ICU population [9, 10]. Importantly, current opinion of the merit of CHG bathing to reduce hospital acquired infections, among adults admitted to intensive care, is divergent [8, 9], suggesting a state of ‘clinical equipoise’ [11].

A challenge when assessing the growing evidence of the effectiveness of an intervention, is that meta-analysis of accumulating data may obtain spurious statistical significance [12–14]. This is thought to be due to an aggregate sample size from the accumulated published trials, lower than that expected to adequately assess effectiveness, may underestimate effect [15]. In an attempt to overcome these potential pitfalls, a Trial Sequential Analysis (TSA) approach has been developed that attempts to address Type I and Type II error, with repeated significance testing of accumulating trial data [16]. Therefore, this TSA meta-analysis was undertaken to summarize the current status of the evidence for the effectiveness of daily CHG bathing, among adult intensive care patients, to reduce various infections in the ICU; and, address the question - should we continue to attempt to assess effectiveness with further trials, or is current evidence adequate to recommend CHG bathing become common practice in the adult ICU?

## Materials and methods

This meta-analysis was planned, undertaken, and has been reported using the preferred reporting items for systematic reviews and meta-analyses (PRISMA) guidelines [17].

### Data sources and search strategy

A systematic literature search was undertaken of medical literature databases including MEDLINE, EMBASE and Cochrane Library published up until March 2017. Keywords and title searches included a combination of: “Chlorhexidine”, “bath$”, “intensive care”, “prevention”, “infection$”, “effectiveness”. Hand searching of the references of research papers was also undertaken until no new studies were identified.

### Study selection

#### Inclusion and exclusion criteria

The eligibility for inclusion of research papers into this meta-analysis was considered independently by two authors (SF and JL). Due to a previous systematic review showing an extreme level of heterogeneity among before-and-after trials, only randomised trials of the effectiveness to reduce infections in adult ICU patients were included. Review papers, non-adult populations, non-ICU, papers that did not report the rates per ICU-days at risk, and trials using CHG bathing combined with other interventions, were excluded.

#### Data extraction and synthesis

Data extracted from each paper included: first author’s name and publication year, country of study, duration of study, study site, study design, type of ICU setting (surgical, medical, and mixed etc.), infection of interest, and number of events and ICU days at risk.

#### Statistical methods

Individual study and combined estimates of the effectiveness of chlorhexidine bathing to prevent infections (Incidence Rate Ratios (IRR)) are presented as forest plots [18]. Both fixed effect - using the Mantel-Haenzel (MH) method and random effect (RE), using the method suggested by Der-Simonian and Laird (DL) [19]) summary estimates are presented. Heterogeneity of effectiveness between studies was assessed using the I^2^ statistic, which was used to estimate the optimal sample size required to address effectiveness, referred to as Information Size (IS), or more specifically the Heterogeneity adjusted Information Size (HIS) in the presence of an assumption of random-effects (I^2^ > 0) [14, 16]. Heterogeneity adjusted Information Sizes were calculated as follows:

$$ \mathrm{HIS}=\mathrm{n}/\left(1-{\mathrm{I}}^2\right) $$

Where, *n* is the sample size estimated for a single trial with adequate power (in our case 0.8), and minimal risk of Type I error (0.05). This sample size is then inflated to address heterogeneity among accumulating data from multiple trials using the I^2^ statistic from a meta-analysis of current published literature [14]. Sample sizes for a single optimal trial was estimated using the methods suggested by Reich et al [20], these methods allow sample size calculation of randomised cluster cross-over trials. Specifically, we used a similar design to that of Noto et al [21], five clusters, of approximately 500 patients, with four cross-over periods – Type I and Type II error set at 0.05 and 0.2, respectively. Baseline risk among control groups, were obtained from meta-analysis of published trials, an inverse-variance method was used to obtained baseline rates (%) among control groups. Upper (superiority), and lower (inferiority) monitoring boundaries (alpha spending) for TSA were estimated using the methods suggested by O’Brien and Fleming [22], and futility boundaries (beta-spending) were also estimated using the *gsDesign* and *ldbounds* packages from R-statistical language [23]. Cumulative *z*-statistics were derived using the methods suggested by Miettinen and Nurminen [24].

## Results

### Search results

**Fig. 1:**
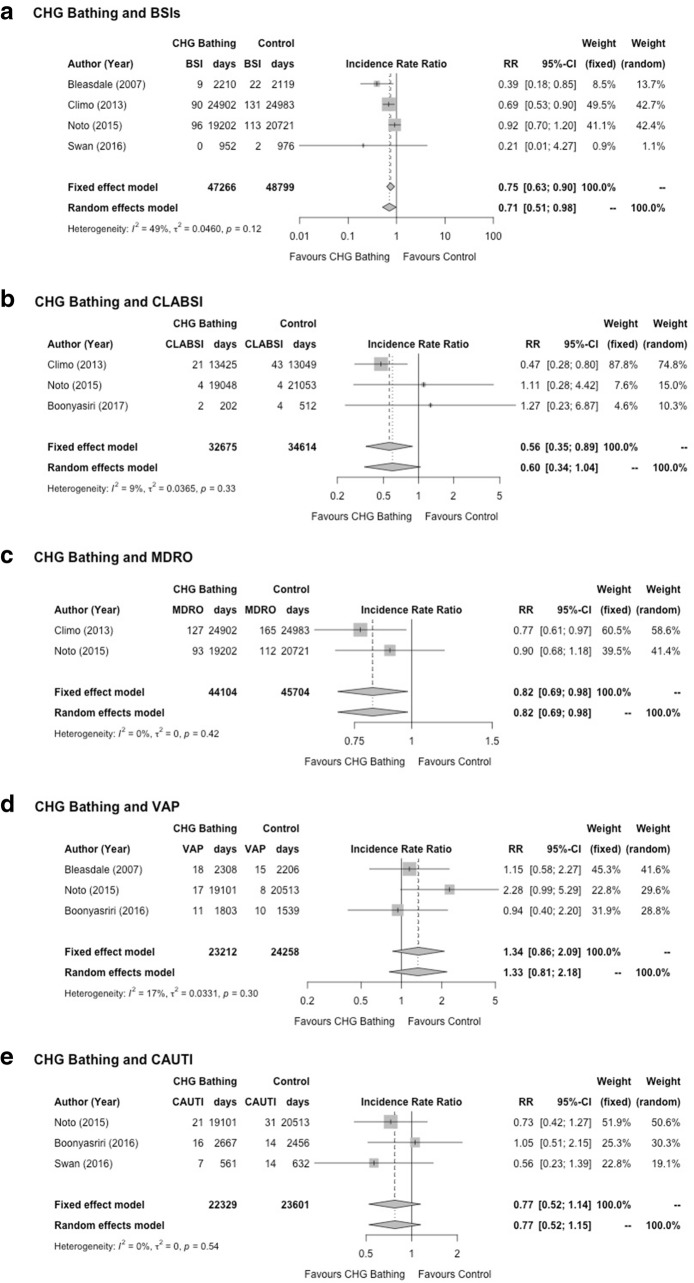
PRISMA Flow diagram of study selection

**Fig. 2:**
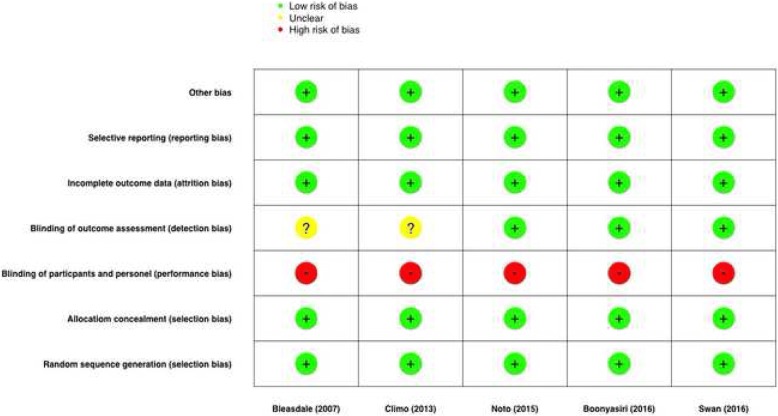
Forest plots of trials assessing effectiveness of CHG bathing to reduce various infection among adults admitted to the intice care. BSI = Blood Stream Infection, CLABSI = Central Line Associated Blood Stream Infection, MDRO = Multi-Drug Resistant Organism, VAP = Ventilator Associated Pneumonia, and CAUTI – Catheter Associated Urinary Tract Infections. CI = confidence interval. Fixed effect estimates are using the Mantel-Haenzel (MH) method and random effect (RE), using the method suggested by Der-Simonian and Laird (DL) [19])

**Fig. 3:**
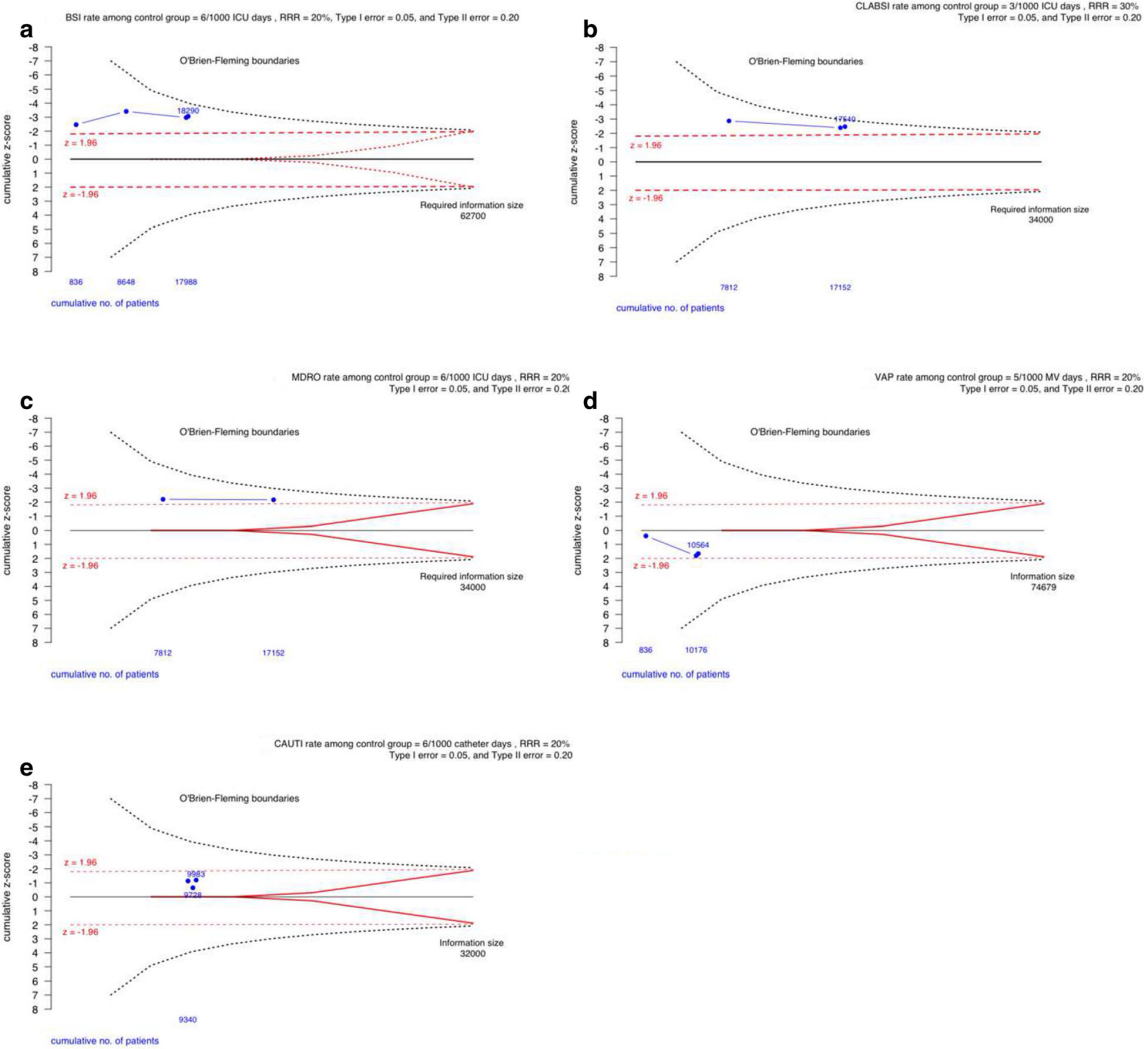
Risk of bias plot

The electronic search resulted in 164 potential papers to be included. Following review of the abstract, or the complete paper when required, using our inclusion and exclusion criteria, 159 papers were excluded (including seven before-and-after studies, and 16 trials among paediatric ICU patients, Fig. 1) - leaving two randomised control, and three randomised-cluster-crossover trials for final analysis. Characteristics of the five randomised trials are presented in Table 1. Summary estimates of the effectiveness CHG bathing to reduce infections among adult ICU patients are presented in Table 2, and in the forest plot figures (Fig. 2). A summary of potential risk of bias for the included studies is presented in Fig. 3.

**Table 1:** Summary of study characteristics of the five trials on daily CHG bathing of ICU patients

First author (year)	Characteristic	Description
Bleasdale, 2007 [27]	Duration	12 months
	Country, ICU setting	USA, medical ICU
	CHG bathing method	Impregnated Cloths
	Outcome(s)	BSI, VAP and c-diff
	Study design	Randomised cross-over
Climo, 2013 [6]	Duration	12 months
	Country, ICU setting	USA, nine ICUs, medical, surgical, cardiac and bone morrow transplant
	CHG bathing method	Impregnated Cloths
	Outcome(s)	BSI, CLABSI, CAUIT and VAP
	Study design	Randomised cross-over
Noto, 2015 [21]	Duration	12 months
	Country, ICU setting	USA Five ICUs
	CHG bathing method	Impregnated Cloths
	Outcome(s)	CLABSI, CAUTI, VAP, and C-diff
	Study design	Randomised cross-over
Boonyasiri, 2016 [28]	Duration	24 months
	Country, ICU setting	Thailand, four ICUs, medical
	CHG bathing method	Impregnated Cloths
	Outcome(s)	CLABSI, VAP, and CAUTI
	Study design	Individual Randomised-Controlled
Swan, 2016 [29]	Duration	12 months
	Country, ICU setting	USA one surgical ICU
	CHG bathing method	Water and diluted CHG
	Outcome(s)	BSI, CLABSI, CAUTI, and VAP
	Study design	Individual Randomised-Controlled

**Table 2:** Summary of specific outcomes of hospital acquired infections among adults admitted to intensive care, from randomised controlled trials

Outcome of interest	no. of trials [ref] (no. of patients)	Summary estimate, Risk Ratio (95% CI) [test of heterogeneity, *p*-value]	Baseline risk (among control period)	Estimated information size^1^
BSI	4-trials [6, 21, 30] (*n* = 18,290)	MH (FE) RR = 0.75 (0.63, 0.91) [I^2^ = 49%, het test, *p* = 0.117]	6 / 1000 ICU days	62,700 (HIS)
CLABSI	3-trials [6, 21, 28] (*n* = 17,540)	MH (FE) RR = 0.56 (0.35, 0.89) [I^2^ = 9%, het test, *p* = 0.331	3 / 1000 lines days	62,700 (HIS)
MRDO	2-trials [6, 21] (*n* = 17,152)	MH (FE) RR = 0.82 (0.69, 0.98) [I^2^ = 0%, het test, *p*-value = 0.416]	6 / 1000 ICU days	34,000 (IS)
VAP	3-trials [21, 30] (*n* = 10,564)	MH (FE) RR = 1.55 (0.79, 3.01) [I^2^ = 17%, het test, *p*-value = 0.213]	5 / 1000 MV days	40,950 (HIS)
CAUTI	3-trials [21, 28, 29] (*n* = 9983)	MH (FE) RR = 0.77 (0.52, 1.14) [I^2^ = 0%, het test, *p*-value = 0.539)	6 / 1000 catheter days	32,000 (IS)

Note: *BSI* Blood Stream Infection, *CLABSI* Central Line Associated Blood Stream Infection, *MDRO* Multi-Drug Resistant Organism, *VAP* Ventilator Associated Pneumonia, and *CAUTI* Catheter Associated Urinary Tract Infections, *CI* confidence interval, *MH* Cochrane-Mantel-Haenszel, *FE* Fixed Effect. Heterogeneity Information size estimated using the approach suggested by Thorlund et al [14]. *HIS* Heterogeneity adjusted Information Size (I^2^ < 0%), *IS* (Information Size, I^2^ > 0%)

### Blood stream infections (BSI)

**Fig. 4:**
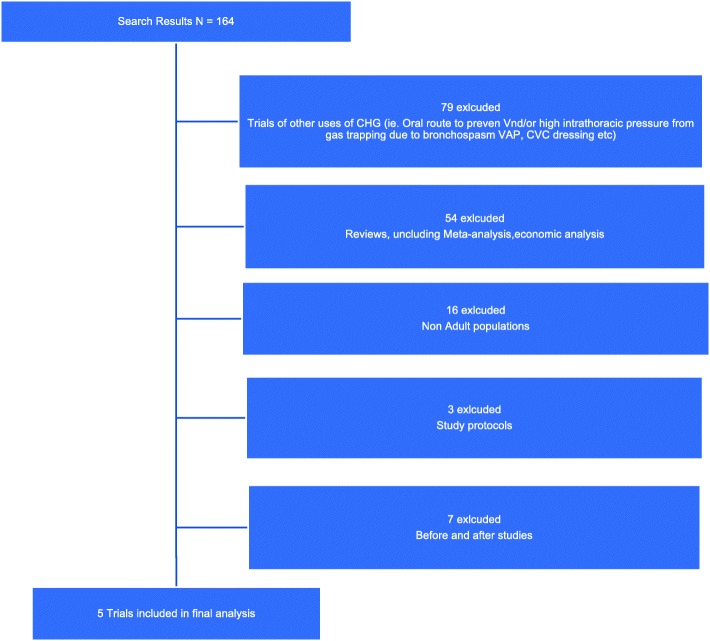
Trial sequential analysis of evidence for the effectiveness of chlorhexidine (CHG) bathing, in adult intensive care patients, to reduce infection. BSI = Blood Stream Infection, CLABSI = Central Line Associated Blood Stream Infection, MDRO = Multi-Drug Resistant Organism, VAP = Ventilator Associated Pneumonia, and CAUTI – Catheter Associated Urinary Tract Infections. RRR = Risk rate reduction. Estimates above the upper boundary (broken line) suggest superiority, while those below the lower boundary, suggest inferiority of CHG-bathing to prevent infection. The required heterogeneity information size estimated using the approach suggested by Thorlund et al [14]

Four randomised trials assessed BSI as a primary outcome of interest (18,290 patients), daily bathing with CHG was estimated to reduce BSI in the ICU by approximately 29% (DL-RE IRR = 0.71, 95% confidence interval (CI) 0.51, 0.98), I^2^ = 49%, *p*-value for heterogeneity = 0.12 (Fig. 2a, and Table 2). TSA of the effectiveness of CHG bathing to reduce BSI among the adult ICU population is presented in Fig. 4a. Even though traditional significance levels have been crossed for superiority (z > 1.96) cumulatively for all four trials, TSA upper O’Brien-Fleming monitoring boundaries have not.

### Central line associated blood stream infections (CLABSI)

Three randomised trials assessed CLABSI as a primary outcome of interest (17,540 patients), daily bathing with CHG was estimated to reduce CLABSI in the ICU by approximately 40% (DL-RE IRR = 0.60, 95% confidence interval (CI) 0.34, 1.04), I^2^ = 9%, *p*-value for heterogeneity = 0.33 (Fig. 2b, and Table 2). TSA of the effectiveness of CHG bathing to reduce CLABSI among the adult ICU population is presented in Fig. 4b. Even though traditional significance levels have been crossed for superiority (z > 1.96) in both cumulative analysis of trials, TSA upper O’Brien-Fleming monitoring boundaries have not been crossed.

### Multi-drug resistant organisms (MDRO)

Two randomised trials assessed MDRO (both studies based on colonization or infection) as a primary outcome of interest (17,152 patients), daily bathing with CHG was estimated to reduce MDRO in the ICU by approximately 18% (DL-RE IRR = 0.82, 95% confidence interval (CI) 0.69, 0.98), I^2^ = 0%, *p*-value for heterogeneity = 0.42 (Fig. 2c, and Table 2). TSA analysis of the effectiveness of CHG bathing to reduce MDRO among the adult ICU population is presented in Fig. 4c. Even though traditional significance levels have been crossed for superiority (z > 1.96) for cumulative analysis of both studies, the TSA upper O’Brien-Fleming monitoring boundaries have not.

### Ventilator associated pneumonia (VAP)

Four randomised trials assessed VAP as a primary outcome of interest (10,564 patients), daily bathing with CHG was estimated to have no effect in reducing VAP in the ICU (DL-RE IRR = 1.33, 95% confidence interval (CI) 0.81, 2.18), I^2^ = 17%, *p*-value for heterogeneity = 0.30 (Fig. 2d, and Table 2). TSA analysis of the effectiveness of CHG bathing to reduce VAP among the adult ICU population is presented in Fig. 4d. Traditional significance levels have not been crossed for superiority (z < 1.96), and therefore the TSA upper O’Brien-Fleming monitoring boundaries have not been crossed also.

### Catheter associated urinary tract infections (CAUTI)

Three randomised trials assessed CAUTI as a primary outcome of interest (9983 patients), daily bathing with CHG was estimated to have no effect in reducing CAUTI in the ICU (DL-RE IRR = 0.77, 95% confidence interval (CI) 0.52, 1.15), I^2^ = 0%, *p*-value for heterogeneity = 0.54 (Fig. 2e, and Table 2). TSA analysis of the effectiveness of CHG bathing to reduce CAUTI among the adult ICU population is presented in Fig. 4e. Traditional significance levels of cumulative trials have not been crossed for superiority (z < 1.96), and therefore the TSA upper O’Brien-Fleming monitoring boundaries have not been crossed also.

## Discussion

This trial sequential meta-analysis presents a summary of the current status of the estimated effectiveness of CHG-bathing to prevent infection among adults admitted to the intensive care. Routine bathing with CHG does not occur in the ICU setting, and TSA suggests that more trials are needed to address the current state of ‘clinical equipoise’. These future studies need to be conducted among a diverse group of ICU patients, including both surgical, medical and trauma patients if possible, to ensure generalisability of results to the majority of patients cared for in the ICU setting.

Previous reviews of daily CHG-bathing to reduce infections among adults admitted to the ICU have been undertaken [7, 10, 25, 26], and suggest a benefit in CHG-bathing to reduce various hospital acquired infections. However, divergent results from two large, well planned and conducted, randomised cluster cross-over trials have not resulted in the widespread adoption of CHG bathing by nurses in the ICU. Specifically, the considerable variation in the baseline risk of infection in the ICU populations included in published trials, to date, has made generalisation difficult.

Our assessment of the current status of evidence of the effectiveness of CHG bathing to reduce the risk of infection in the intensive care suggests that more research in this area is needed. Specifically addressing two important short comings of current evidence, namely: (1) future trials need to include a diverse population of adults admitted to the ICU; and, (2) future trials need to be powered to add to the current cumulative data, to ensure clear statistical evidence of benefit, and avoid spurious results due to Type I error.

A potential strength of our meta-analysis is the use of TSA to avoid a spurious conclusion of the effectiveness of CHG bathing, and to address what needs to be done next due to the current ‘clinical equipoise’ of this intervention in the ICU setting. However, any systematic review and meta-analysis have a potential weakness of missing unpublished trials, and potential individual trial heterogeneity that is difficult to account for in analysis. We purposely omitted before–and-after trials that tend to overestimate effectiveness and contained significant within-trial-variation [10], adding these trials would significantly increase the respective cumulative samples size, but would not add quality to any estimates of effect. A potential limitation of our meta-analysis is that mortality outcomes between CHG-bathing and comparison groups was only reported by two trials, and such an outcome has an important place in describing the ultimate burden of hospital acquired infection in the ICU setting. And, our meta-analysis lacks the data to answer the question – Do we need to bath all patients, or only those at the greatest risk, or already colonized? Further to this, the study by Boonyasrir et al [27] didn’t use prepacked CHG impregnated clothes, instead CHG wash cloths were prepared by staff at the bedside, therefore the dose of CHG may have been unstandardized.

The lack of evidence for the benefit of daily CHG-bathing reported by Noto et al [21] and positive results reported by Climo et al [6], have been suggested to be due to a short ten-week intervention and control periods used in the study by Noto [21], compared to the 6-months cross-over period used by Climo [6]. The shorter ten-week cross-over period being considered to be insufficient to determine the true impact of CHG bathing on infection rates. Therefore, future trials should consider the optimal study design, and the optimal length of study intervention and control periods. The use of a randomised cluster cross-over design appears optimal and would be easily integrated into the current cumulative evidence. However, the contextual effect of the intervention period lowering the background rates of infection would need to be carefully considered, and for this reason a randomised stepped-wedge approach may also be an option [28].

The results of our TSA meta-analysis have some important clinical implications for the wider ICU community of clinicians. Importantly, more research is need in this area, that specifically ensures trustworthy evidence of the effectiveness of daily CHG bathing to reduce infections among adults admitted to intensive care, and to ensure results are generalisable to a wider diverse population of ICU patients. The evidence needs to be of the highest quality, like any intervention, there are concerns regarding the safety of daily bathing of ICU patients with CHG, however our meta-analysis has not addressed this issue. Moreover, even though CHG bathing aims to reduce HAI, it has been suggested it may promote the emergence of chlorhexidine-resistance, and increase gram-negative organism bacteraemia [9]. However, large studies have failed to support this hypothesis [29]. Importantly, even a modest treatment effect should be considered in the context of the seriousness of some of these specific infections among ICU patients, that are costly and are potentially associated with increased patient morbidity and mortality.

Importantly, a previous met-analysis of CHG bathing by Afonso et al [30] found that the effect of chlorhexidine gluconate-impregnated washcloth bathing may be unequal for Gram-positive BSIs versus Gram-negative BSIs and that this warrants further study.

## Conclusion

Routine bathing with CHG does not occur in the adult ICU setting, and TSA suggests that more trials are needed to address the current state of ‘clinical equipoise’. Ideally these studies would be conducted among a diverse group of ICU patients, and to the highest standard to ensure generalisability of results.

## Abbreviations

BSI: Blood Stream Infection
CAUTI: Catheter Associated Urinary Tract Infections
CHG: Chlorhexidine Gluconate
CI: Confidence Interval
CLABSI: Central Line Associated Blood Stream Infection
HAI: Health Care Acquired Infection
ICU: Intensive Care Unit
IRR: Incidence Rate Ratio
RE: Random Effects
VAP: Ventilator Associated Pneumonia
